# Flower within the Crannied Walls

**Published:** 1903-04

**Authors:** 


### FLOWER WITHIN THE CRANNIED WALLS.

The troubles of "Dr." R. C. Flower, which have been ventilated freely in the newspapers, will be read with appreciative interest by numbers of people in Atlanta. Flower belonged to the spellbinder type of professional grafter. He cured by laying on of hands, a sort of magnetic touch, the influence of which was especially noticed in 'the attraction (and extraction) of silver and gold disks bearing the image and superscription of the great republic. The coins responded to the magic attraction with a leap that transferred them from the pocket of the patient to that of the doctor—and it was irresistible—like the nails drawn from the ship by the magnetic mountain in the tale of Sinbad the Sailor,

Flower was not the wee tippet kind, nor the sort that is born to blush unseen. It is very likely that on the contrary, he was never seen to blush, the blushing was done by others, and after his departure. It will be remembered that some years ago the Atlanta newspapers were all agog over Flower's great scheme to establish a vast sanitarium sufficient to house all the sick in the state, who needed the healing touch to rid them of their ills. But the scheme flashed in the pan, and the flower dried up on the stem. Of late years, he seemed to have forgotten the over worked magnetic healing graft, and turned his genius into the allied industry of working the unwary with fake mines and oil stock, and such like speculative gold brick schemes that flourish on the gullibility of the public.

Now Flower, possibly from the surplusage of moisture in his stocks has been converted into dough, and is being kneaded and flattened between the rolling pins of judicial inquiry, and will in all likelihood be dried to the consistency of a soda cracker and mould in duress for a term of years. Truly, man springeth up like a flower and is cut down.

But one cannot suppress a contemptuous admiration for Flower. He was in a sense great. His was a masterful spirit. He understood human nature and lived off its weakness, because he was wiser than most of his kind. The wickedness of the world was print to him," That he landed in jail is a natural sequence, but for all that, he was an uncommon man for all he was such a rascal.

TO PREVENT INFECTION. A practical and helpful series of rules for the sanitary management of contagious and infectious diseases has been prepared by The Palisade Manufacturing Company of Yonkers, and issued in pad form with cover.

It is intended that when called to a contagious case the physician shall sign and hand to the attendant one of these printed sheets of "Precautions to be Observed by Patient, Family and Attendants." This series of rules, couched in plain, every day English, has been carefully prepared and the information given is accurate and up-to-date. The delivery of such a signed code of instructions not only impresses the family favorably, but also relieves the physician of all responsibility should any of the necessary precautions be omitted. The advertising of Borolyptol is so arranged that, if the physician desires, he can detach all reference to the preparation before handing the directions to the family.

One of these pads (thirty-two sheets) will be mailed to any physician who may apply for same.

